# 

**Published:** 1901-09-15

**Authors:** 


### ANNOUNCEMENTS.

### AMERICAN SOCIETY OF ORTHODONTISTS.

At the first annual meeting of the American Society of Orthodontists, held in St. Louis, Mo., the following officers were elected: President, Edward H. Angle, St. Louis; Vice President, William J. Brady, Iowa City; Secretary-Treasurer, Milton T. Watson, Detroit. Board of Censors, Richard Summa, St. Louis; Henry E. Lindas, Great Bend, Kansas; William E. Walker, New Orleans.

MILTON T. WATSON,

*Secretary.*

### NATIONAL ASSOCIATION OF DENTAL FACULTIES.

At the annual meeting, held at Milwaukee, August 1 to 6, 1901, the following officers were elected: President, W. F. Litch; Vice President, G. V. I. Brown; Secretary, J. H. Kennerly; Treasurer, H. W. Morgan. Executive Committee, S. W. Foster, H. B. Tileston, D. J. McMillen, J. I. Hart, J. B. Wilmott. *Ad Interim* Committee, J. P. Gray, W. T. McLean, A. H. Peck. Committee on Law, H. W. Morgan, W. A. Montelle, F. D. Weisse.

### NATIONAL SOCIETY OF DENTAL EXAMINERS.

At the annual meeting, held at Milwaukee, August 2 to 6, 1901, the following officers were elected: President, J. F. Dowsley; Vice President for East, C. A. Meeker; Vice President for South, J. A. Hall; Vice President for West, B. L. Thorpe; Secretary-Treasurer, J. Allen Osmun.

### NATIONAL DENTAL ASSOCIATION.

At the annual meeting of the National Dental Association, held at Milwaukee, August 6 to 9, 1901, the following officers were elected for the ensuing year: President, J. A. Libbey, Pittsburg; Vice President for East, S. H. Guilford, Philadelphia; Vice President for South, L. G. Noel, Nashville; Vice President for West, W. P. Dickinson, Minneapolis; Recording Secretary, A. H. Peck, Chicago; Corresponding Secretary, Josephine D. Pfeifer, Chicago; Treasurer, H. W. Morgan, Nashville. Executive Council, H. J. Burkhart, Batavia, N. Y.; B. Holly Smith, Baltimore; J. Y. Crawford, Nashville; C. C. Chittenden, Madison, Wis.; M. F. Finley, Washington. Executive Committee, J. D. Patterson, Kansas City; C. S. Butler, Buffalo; C. N. Johnson, Chicago; H. A. Smith, Cincinnati; V. H. Jackson, New York; T. P. Hinman, Atlanta; T. S. Waters, Baltimore; W. N. Cogan, Washington; G. V. I. Brown, Milwaukee. The next meeting will be held at Niagara Falls in August, 1902.

### FOOD OF PREHISTORIC MAN.

Our attention has recently been called to some curious experiments conducted some time ago by Mr. Charters White, M. R. C. S., lately the president of the Royal Odontological Society of Great Britain. Upon examining some skulls dating back from the stone age, he noted that several of the teeth, although quite free from caries, were thickly coated with tartar. It occurred to him that it would be possible by a rough analysis to identify any particles of food that might be embodied in this natural concrete, and so reveal the character of the aliment partaken of by prehistoric man. Dissolving the tartar in weak acid, a residue was left which, under the microscope, was found to consist of cornhusk particles, hairs from the outside of the husks, spiral vessels from vegetables, particles of starch, the point of a fish tooth, a conglomeration of oval cells probably of fruit, the barblets of down and portions of wool. In addition to this varied list were some round, red bodies, the origin of which defied detection, and many sandy particles, some relating to quartz and some to flint. These mineral fragments were very likely attributable to the rough stones used in grinding the corn, and would account for the erosion of the masticating surfaces, which in many cases was strongly marked. This inquiry into the food of men who lived not less than 4,000 years ago is a matter of great archæological interest.—*Chambers' Journal.*

